# Photoperiod Control of Plant Growth: Flowering Time Genes Beyond Flowering

**DOI:** 10.3389/fpls.2021.805635

**Published:** 2022-02-09

**Authors:** Michela Osnato, Ignacio Cota, Poonam Nebhnani, Unai Cereijo, Soraya Pelaz

**Affiliations:** ^1^Centre for Research in Agricultural Genomics, CSIC-IRTA-UAB-UB, Barcelona, Spain; ^2^Institute of Environmental Science and Technology of the Universitat Autònoma de Barcelona, Barcelona, Spain; ^3^Institució Catalana de Recerca i Estudis Avançats, Barcelona, Spain

**Keywords:** photoperiod, growth, flowering, stomata, adaptation, growth cessation, tuberization, runner

## Abstract

Fluctuations in environmental conditions greatly influence life on earth. Plants, as sessile organisms, have developed molecular mechanisms to adapt their development to changes in daylength, or photoperiod. One of the first plant features that comes to mind as affected by the duration of the day is flowering time; we all bring up a clear image of spring blossom. However, for many plants flowering happens at other times of the year, and many other developmental aspects are also affected by changes in daylength, which range from hypocotyl elongation in *Arabidopsis thaliana* to tuberization in potato or autumn growth cessation in trees. Strikingly, many of the processes affected by photoperiod employ similar gene networks to respond to changes in the length of light/dark cycles. In this review, we have focused on developmental processes affected by photoperiod that share similar genes and gene regulatory networks.

## Introduction

The Earth’s rotation on its axis and revolution around the sun create cycles of day and night with a 24-h period as well as changes in temperature and humidity with a 365-day period. These periodic alterations in environmental conditions (light duration and intensity, ambient temperature) are modest in tropical and subtropical regions, where changes in precipitation determine the two main seasons (e.g., dry and wet), but are considerable in temperate regions, where changes in the length of the day and ambient temperature determine the four seasons – **Long Days** (LDs) in spring and hot summer, and **Short Days** (SDs) in autumn and cold winter.

Compared to temperature, daylight is a more predictable external cue that enables organisms to anticipate seasonal changes and modulate their biological function consequently. The length of the light period over 24 h, also named **photoperiod**, regulates many aspects of plant growth. For example, trees stop growing when days shorten in autumn foreseeing the arrival of cold winter. There are more examples of developmental traits regulated by changes in photoperiod, some discussed in this review. Their importance in plant development and plant adaptation to specific habitats promoted scientific research about the molecular mechanisms underlying photoperiodic response.

Light/dark cycles and hot/cold cycles entrain the **circadian clock**, defined as the internal timer synchronized with solar time that oscillates with a stable phase of approximately 24 h.

Light is perceived by photoreceptors that sense different wavelengths of the natural sunlight spectrum (reviewed in Lin, 2000), which then transmit this information to the central oscillator of the circadian clock – made of interconnected molecular gears that generates a 24 h rhythm. Components of the circadian clock are encoded by regulatory genes that are activated at specific time points, such as morning-phased genes at the beginning of the light period or evening-phased genes at the beginning of the dark period. These regulatory proteins form multimeric complexes, which act in multiple feedback loops that in turn affect the expression of downstream targets at different moments, from sunrise to sunset and during the night.

Genetic determinants underlying light perception and the mechanism of the clock have been extensively characterized in the model species *Arabidopsis thaliana*, and then identified in other plants such as rice and potato.

In Arabidopsis, five phytochrome family members (phyA to phyE) represent the major photoreceptors that sense red and far-red light (Clack et al., 1994). Phytochromes are localized in the cytosol in their inactive form but are translocated to the nucleus in their active forms, where they associate with different regulatory proteins, including the PHYTOCHROME INTERACTING FACTORS (PIFs, Matsushita et al., 2003).

Genes encoding the MYB-like transcription factors CIRCADIAN CLOCK-ASSOCIATED 1 (CCA1) and LATE ELONGATED HYPOCOTYL (LHY) are expressed at dawn (Schaffer et al., 1998; Wang and Tobin, 1998). CCA1 and LHY proteins interact to form the morning complex, which regulates the expression of genes encoding PSEUDO-RESPONSE REGULATOR (PRR) proteins (Alabadí et al., 2001; Farré et al., 2005; Lu et al., 2009; Kamioka et al., 2016). In particular, CCA1-LHY repress the key element of the central oscillator *PRR1/TIMING OF CAB EXPRESSION* 1 (TOC1) at the beginning of the light period (Alabadí et al., 2001; Nakamichi et al., 2010; Gendron et al., 2012). Other *PRR* genes peak sequentially throughout the day: *PRR9* at dawn, PRR7/PRR5/PRR3 in the afternoon and TOC1 at dusk (Nakamichi et al., 2005a,b). During the night, TOC1 represses the expression of *CCA1* and *LHY* (Alabadí et al., 2001).

The morning complex also represses the expression of genes encoding components of the evening complex such as GIGANTEA (GI, Fowler et al., 1999; Berns et al., 2014), EARLY FLOWERING 3 (ELF3, Hicks et al., 1996; Covington et al., 2001) and *ELF4* (Doyle et al., 2002). The evening complex suppresses *PRR9*, restricting its expression to the early morning (Chow et al., 2012).

In summary, light perception triggers a cascade of molecular events that activate circadian clock associated genes at different times of the day (Pokhilko et al., 2012). Also, seasonal fluctuations in external conditions represent the input information that adjusts the circadian clock year-round. Thus, this mechanism measures the length of the daylight (and ambient temperature) to trigger seasonal responses. Ultimately, the clockwork regulates the diurnal oscillations of the transcription of many output genes that control several developmental and physiological processes underlying the adaptation of an organism – animals and plants – to a changing environment (reviewed by Nohales and Kay, 2016).

In plants, photoperiod integrates the circadian clock output information and the light perception to regulate plant adaptation to different light regimes. Accordingly, plants modulate their growth in response to seasonal changes and synchronize key developmental transitions with favorable conditions to sustain their ecological fitness (i.e., survival of adult organisms able to produce progeny before their death). Different photoperiods regulate different developmental transitions; for example, in poplar trees LDs trigger vegetative growth in spring while SDs promote growth cessation in autumn. On the other hand, the same developmental process could be regulated by different photoperiods in different plants; a good example is the transition from the vegetative to reproductive phase (also called the **floral transition**), because some plants growing in temperate regions flower when days are long whereas other plants growing in tropical regions prefer SD or even neutral days (12 h light/12 h dark) to develop reproductive structures. Hence, photoperiod controls several developmental stages throughout the plant life cycle.

In this review, we focus on processes that share genes and gene regulatory networks (GRNs) rather than those that are regulated by completely different mechanisms or without detailed molecular/genetic studies.

## Photoperiodic Control of Hypocotyl Elongation in Arabidopsis

Upon seed germination, the elongation of the hypocotyl (i.e., the stem of a germinating seedling in dicotyledonous plants) is one of the earliest processes affected by photoperiod. Molecular mechanisms underlying this developmental step have been extensively studied in Arabidopsis, which displays short hypocotyls when grown under LDs but long hypocotyls under SDs.

The regulation of hypocotyl growth relies on the activity of PIF4 and PIF5, two basic helix-loop-helix (bHLH) transcription factors which protein abundance is controlled by the photoreceptor phyB that mediates PIFs degradation during the day through the 26S proteasome (Huq and Quail, 2002). Several studies indicate that the hypocotyl elongates in the dark period, consistent with higher accumulation and activity of PIFs before dawn (Nozue et al., 2007).

Under SD, PIFs promote hypocotyl growth at the end of the long night, when they form dimers and bind to regulatory elements of target genes involved in cell expansion and auxin signaling (Khanna et al., 2004; Kunihiro et al., 2011; Hornitschek et al., 2012; Zhang et al., 2013). PIFs also activate CYCLING DOF FACTOR 1 (CDF1) and CDF5, two transcription factors belonging to the DNA-binding with One Finger (DOF) family, that in turn promote the expression of *YUCCA8* (*YUC8*), an important auxin biosynthetic gene (Martín et al., 2018).

Components of the circadian clock also regulate the daily oscillation in *PIFs* expression and PIF protein stability. The Evening Complex in association with PRRs represses PIF4/PIF5 transcription until early night (Matsushika et al., 2000; Nakamichi et al., 2005a,b; Nusinow et al., 2011). Unlike PIFs, PRRs repress the expression of *CDFs* during the day, thus preventing hypocotyl elongation in the light period (Martín et al., 2018).

Hypocotyl elongation is also controlled by the phytohormone Gibberellic Acid (GA) through the degradation of DELLA proteins, negative regulators of growth able to interact with PIFs. During the light period, DELLA protein levels are high and sequester PIFs: the formation of inactive complexes hinders PIF binding to target genes. During the dark period, GA accumulation triggers DELLA degradation, allowing the release of PIFs. Thus, PIFs accumulate in the nucleus at night and activate downstream genes involved in hypocotyl growth before dawn (Davière et al., 2008; De Lucas et al., 2008; Feng et al., 2008).

## Photoperiodic Control of Seasonal Flowering in Annual Plants

Besides germination and seedling establishment, the transition from the vegetative to the reproductive phase represents a critical step for the reproductive success of flowering plants. Floral induction must occur at the most favorable season to guarantee the highest production of seeds and survival of the offspring. This phase change is economically relevant in seed crops as it determines grain yield, an important agronomic trait. Accordingly, both precocious and delayed flowering should be avoided as they might cause yield losses. Early flowering plants fail to store enough energy for seed development due to insufficient growth of photosynthetic organs; conversely, late flowering plants are vigorous, but fertility can be affected if seed maturation takes place in adverse weather conditions. Therefore, plants should synchronize leaf and flower development with environmental conditions to optimize the timing of the formation of reproductive organs.

Multiple genetic determinants control the switch from vegetative to reproductive growth by integrating external cues and internal signals. Before floral transition, the plant undergoes the juvenile-to-adult transition when it reaches the competence to flower in response to inductive environmental conditions. Although photoperiod is one of the major environmental stimuli, others such as ambient/seasonal temperatures together with endogenous conditions (e.g., plant age, accumulation of sugars, and hormones) contribute to finely tune the floral induction.

Control of flowering time has been massively studied in *Arabidopsis thaliana*. Nevertheless, research conducted in many other species - including monocotyledonous plants - has shown that GRNs controlling the floral transition are mostly conserved, albeit with some variations that account for the peculiarities of the different plant species examined.

### Photoperiodic Flowering Under Long Days in the Model Species Arabidopsis

Arabidopsis is a facultative LD plant, meaning that although it can bloom in SDs it flowers much more rapidly when days are long (e.g., 16 h light/8 h dark). The length of the day is sensed in the leaf, but the floral induction takes place in the shoot apex (reviewed by Zeevaart, 2009). The transition to flowering involves the existence of a florigen – a mobile signal that travels from the leaf to the Shoot Apical Meristem (SAM) through the phloem (Zeevaart, 1976; Kinoshita and Richter, 2020).

Among hundreds of flowering time genes described to date (Kinoshita and Richter, 2020), *CONSTANS (CO)* is one of the most studied regulators acting in photoperiodic flowering. The expression of the *CO* gene oscillates with the circadian rhythm and is activated in the leaf upon LD perception (Putterill et al., 1995; Suárez-López et al., 2001). CO is only capable to promote flowering under LD, when the peak of *CO* mRNA expression occurring at the end of the daytime coincides with the stabilization of CO protein mediated by light (Suárez-López et al., 2001; Valverde et al., 2004).

Several circadian clock-associated proteins, previously described as regulators of hypocotyl growth, were also found to control CO at transcriptional or post-translational level.

Under LDs, members of the CDF family act as floral repressors by inhibiting *CO* expression in the first part of the light period (Fornara et al., 2009). In particular, CDF1 negatively regulates CO in the morning by interacting with the TOPLESS (TPL) co-repressor (Causier et al., 2012; Goralogia et al., 2017). The combination of multiple mutations in *CDFs* genes leads to photoperiod insensitiveness and early flowering caused by *CO* upregulation (Fornara et al., 2009).

Conversely, PRR5/PRR7/PRR9 act as floral activators by repressing *CDF1* in the afternoon (Nakamichi et al., 2007). Functional PRRs promote flowering under inductive conditions by indirectly activating *CO* expression but they also contribute to the accumulation of CO protein in the light period (Hayama et al., 2017). By contrast, defective PRRs reduce photoperiodic sensitivity: loss of PRR function causes CO downregulation, which leads to late flowering under LD but not under SD (Sato et al., 2002; Yamamoto et al., 2003; Nakamichi et al., 2005a,b, 2007).

In a similar way, the clock associated proteins GI and Flavin-binding Kelch repeat F box 1 (FKF1) positively regulate flowering mostly by preventing the action of repressors on *CO. Actually*, GI and FKF1 form a complex that mediates the ubiquitin-dependent degradation of CDF1 in the late afternoon, resulting in the transcriptional activation of CO at dusk (Imaizumi et al., 2005; Sawa et al., 2007).

In the opposite way, ELF3 and ELF4 negatively regulate flowering by forming different protein complexes that in turn affect the expression of genes of the core and output of the circadian clock (nicely reviewed in Zhao et al., 2021). Interestingly, the evening complex of the circadian clock represses *GI* expression, ELF3 promotes GI degradation, and ELF4 removes GI from the CO promoter (Yu et al., 2008; Kim et al., 2013; Ezer et al., 2017; Park et al., 2019).

Photoreceptors also contribute to the regulation of flowering time (Bagnall et al., 1995). PHYA stabilizes CO whereas PHYB plays the opposite role (Valverde et al., 2004). PHYB also impairs PIF function (Al-Sady et al., 2006; Goyal et al., 2016) that, in addition to their role in hypocotyl elongation, have been shown to integrate light and temperature perceptions. PIF4, PIF5, and PIF7 promote flowering at warm ambient temperatures or in response to the shade avoidance syndrome (Kumar et al., 2012; Fernández et al., 2016; Galvāo et al., 2019; Silva et al., 2020). Interestingly, PIFs are regulated by ELF3; PHYB stabilizes ELF3, and ELF3 interacts with PIF4 and PIF7 to impair the binding to their targets (Xing et al., 2001; Nieto et al., 2015; Jiang et al., 2019).

*CONSTANS* is a B-box (BBX) Zinc Finger transcription factor whose activity is also regulated by protein-protein interactions with other circadian regulated BBX proteins. For example, BBX19 interacts with CO and depletes the active CO pool (Wang et al., 2014), and BBX microProteins miP1a and miP1b likely act as bridge between CO and TPL in a repressor complex which results in the incapability of florigen activation (Graeff et al., 2016).

### The Florigen in Arabidopsis

*CONSTANS* activates the expression of the floral promoter *FLOWERING LOCUS T* (*FT*) in leaf vascular tissue in LDs (Suárez-López et al., 2001; Takada and Goto, 2003; Valverde et al., 2004). The fact that the constitutive expression of *CO* and *FT* confers very early flowering phenotype while their loss of function causes extremely late flowering (but only in LDs) suggested that these two genes might control the first moments of the floral induction in response to photoperiod (Kardailsky et al., 1999; Kobayashi et al., 1999). Nevertheless, the activation of *CO* and *FT* happens in the leaf but not in the SAM in wild-type plants. The circle was rounded when the FT protein was identified as the long-distance signaling molecule able to induce flower development in the shoot apex (Corbesier et al., 2007; Jaeger and Wigge, 2007; Mathieu et al., 2007).

The *FT* gene encodes a small globular protein belonging to the Phosphatidyl Ethanolamine Binding Protein (PEBP) family (Kardailsky et al., 1999; Kobayashi et al., 1999). As FT is not itself able to bind DNA, it interacts with FD, a bZIP transcription factor present at the SAM (Abe et al., 2005; Wigge et al., 2005), and this association is mediated by 14-3-3 proteins (Abe et al., 2019; Collani et al., 2019). The formed regulatory complex - also known as **Florigen Activation Complex** (**FAC**) – first activates *SUPPRESSOR OF OVEREXPRESSION OF CO 1* (*SOC1*) and *APETALA1* (*AP1*), two genes of the *MADS-b*ox family involved in floral meristem identity, by directly binding their promoters (Simon et al., 1996; Samach et al., 2000; Moon et al., 2005; Yoo et al., 2005). Similar to *FT*, the closely related gene *TWIN SISTER OF FT* (*TSF*) is activated by CO and the encoded protein acts as a long-range signal traveling to the SAM where it also interacts with FD (Samach et al., 2000; Yamaguchi et al., 2005; Jang et al., 2009).

Bioactive GAs act as another signal that affects flowering under LDs, but it is under non-inductive SDs that they play a more evident role in floral induction (Mutasa-Göttgens and Hedden, 2009) when CO is almost completely inactive. DELLAs inhibit the action of floral activators through protein interactions, thus affecting transcriptional activation of the floral integrators (FT, TSF, and SOC1) and consequently flowering time under both LDs and SDs (Wang et al., 2016; Xu et al., 2016).

### Negative Regulators of the Floral Transition in Arabidopsis

Floral repressors prevent precocious flowering and guarantee a vegetative phase long enough to accumulate necessary energy reserves for flower and seed production (Boss et al., 2004). Besides the CO repressors CDFs and ELFs, additional factors have been described that directly regulate the florigens (reviewed in Yant et al., 2009; Kinoshita et al., 2020). Among others, two members of the Related to ABI3 and VP1 (RAV) family of transcription factors – named TEMPRANILLO1 (TEM1) and TEM2 – repress flowering under different conditions (Castillejo and Pelaz, 2008; Osnato et al., 2012; Marín-González et al., 2015; Aguilar-Jaramillo et al., 2019), but in the photoperiod pathway TEM proteins counteract CO activity in a quantitative balance to tightly control *FT* expression (Castillejo and Pelaz, 2008). Also, TEMs seem to interact with TPL and TPL-related (TPR) proteins, and this interaction may confer their repressive activity (Causier et al., 2012). However, further experiments are needed to assess this association requirement for TEM function.

In wild-type plants, *TEM* expression levels are high at early stages of vegetative growth but progressively decay to a minimum at the time of floral transition, thus allowing the activation of *FT* by CO (Castillejo and Pelaz, 2008). Thus, *TEM* expression pattern suggests that the balance between TEM and CO activities might be modulating *FT* transcription and consequently adjusting the timing of the floral transition under LD. Besides, as both *CO* and *TEM* are regulated by the circadian clock, they could be acting on *FT* at the same level but antagonistically. Supporting this hypothesis, an impaired balance between the activator and the repressor results in an according variation of the *FT* transcripts levels and alterations of flowering time. In transgenic plants growing in LD, accumulation of the floral activator CO (in overexpression/gain of function lines) has the same effect on flowering as the removal of the floral repressors TEM (in loss of function mutants), obtaining a precocious flowering phenotype. In the same way, plants with reduced levels of both *CO* and *TEM* flower at the same time as wild type plants (Castillejo and Pelaz, 2008).

*CONSTANS* physically associates with NUCLEAR FACTOR Y (NF-Y) proteins, which bind CCAAT sequences present in the *FT* promoter. This binding results in a chromatin loop that brings enhancers present in distal elements close to two CO Responsive Elements (CORE1 and 2) found near the transcription start site of FT (Adrian et al., 2010; Tiwari et al., 2010). NF-Y proteins help recruitment of CO to regulatory sequences in proximal elements that are essential to activate the transcription of *FT* (Cao et al., 2014). Strikingly, TEM proteins recognize a RAV binding site located in the 5′UTR of *FT*, very close to the CORE elements bound by CO (Castillejo and Pelaz, 2008; Cao et al., 2014). This may account for the proposed TEM/CO competition for the *FT* binding sites. Alternatively, TEM binding may affect the *FT* chromatin loop and therefore interfere with its transcriptional activation.

Circadian clock output pathway that promotes photoperiod-dependent flowering comprises the antagonistic CO and TEM activities (Castillejo and Pelaz, 2008). Hence, *FT* levels are the result of a quantitative balance between the respective promoter and repressive activities in the leaf. Nevertheless, *TEM* genes are also expressed in the SAM and their specific downregulation in the shoot apex results in early flowering phenotype, suggesting that *FT* is also repressed in this domain (Osnato et al., 2012). Intriguingly, the *FT* repressive function of CO-miP1a7b-TPL mentioned above (Graeff et al., 2016) seems to be limited to the SAM (Rodrigues et al., 2021). Therefore, *FT* could be actively repressed at the SAM to avoid floral transition before LDs have been sensed in leaves.

The diurnally regulated MADS-domain protein SHORT VEGETATIVE PHASE (SVP) is another important floral repressor (Hartmann et al., 2000; Fujiwara et al., 2008; Jang et al., 2009). At low temperatures, SVP represses *FT* indirectly through activation of *TEM2* (Marín-González et al., 2015) as well as directly through interaction with two related MADS-domain proteins – FLOWERING LOCUS C (FLC) and FLOWERING LOCUS M (FLM).

*FLOWERING LOCUS T* repression is mediated by SVP-FLC during the vernalization process (Li et al., 2008; Mateos et al., 2015) and by SVP- FLM in response to changes in ambient temperature (Lee et al., 2013; Posé et al., 2013). Interestingly, the *FLM* is subjected to alternative RNA splicing that generates two splice forms depending on the external conditions: plants growing in cooler environments accumulate FLMβ, a protein variant that contains the DNA binding domain; plants growing in warmer environments accumulate FLMδ, a protein variant that lacks the DNA binding domain. As a result, the SVP- FLMβ complex delays the floral transition at low ambient temperature by directly repressing *FT*; by contrast, the SVP- FLMδ complex fails to bind FT regulatory regions, thus accelerating flowering at elevated ambient temperature (Lee et al., 2013; Posé et al., 2013).

Instead, loss of function mutations in *SVP* result in early flowering and reduced sensitivity to photoperiod or ambient temperature (Jeong et al., 2007).

The function of the floral repressors TEM and SVP could be prevented by the interaction with the floral activator GI, found to bind sequences of the *FT* promoter in proximity of SVP and TEM binding sites (Sawa and Kay, 2011). Besides CO activation, GI might promote flowering also by impeding accessibility of floral repressors to *FT* regulatory regions and/or by perturbing their repressive activity through physical association (Sawa and Kay, 2011).

SHORT VEGETATIVE PHASE and TEMs also control flowering time by negatively regulating GA biosynthetic genes: SVP represses *GA20OXIDASE2* (*GA20OX2*) (Andrés et al., 2014) while TEM1 directly represses *GA3OXIDASE1* (*GA3OX1*) and *GA3OX2* (Osnato et al., 2012).

Other key regulators of the photoperiod pathway are APETALA2 (AP2) and AP2-related proteins – SCHLAFMUTZE (SMZ), SCHNARCHZAPFEN (SNZ) and TARGET OF EARLY ACTIVATION TAGGED 1 (TOE1), TOE2, and TOE3 – that negatively regulate the floral transition by interacting with TPL (Aukerman and Sakai, 2003; Schmid et al., 2003; Jung et al., 2007; Mathieu et al., 2009; Yant et al., 2010). The expression of these *AP2-like* genes is regulated by microRNA172 (miR172), which independently controls the juvenile to adult transition (Wu et al., 2009) and the floral induction in leaves (Aukerman and Sakai, 2003; Jung et al., 2007; Mathieu et al., 2009).

The synthesis of mature *miR172* relies on five *MIR172* genes (A to E), recently shown to play common and divergent roles under different conditions (Lian et al., 2021; Ó’Maoiléidigh et al., 2021). Specifically, *MIR172*A/B/D also act in the SAM to control the floral transition under SDs (Ó’Maoiléidigh et al., 2021) and *MIR172A/D* under LDs (Lian et al., 2021).

*miR172* abundance is negatively regulated by the floral repressors SVP and TEM. SVP represses *miR172* via direct binding to pri-miR172a (Cho et al., 2012) whereas TEM1 binds a regulatory region of the MIR172C gene. In addition to *MIR172C*, *MIR172A* and *MIR172B* are also downregulated in *tem* double mutants (Aguilar-Jaramillo et al., 2019).

Conversely, *miR172* expression is promoted by GI (Jung et al., 2007), showing that GI regulates flowering time also by affecting *miR172* expression.

Because floral induction is key for plants species survival, the regulatory sequences of genes encoding key floral activators (CO, FT/TSF) have been subjected to natural variation in order to adapt to the environment (Liu et al., 2014; Rosas et al., 2014; Bao et al., 2019).

### Flowering Time Genes and Stomata Functioning in Arabidopsis

Stomata are specialized cell structures that control gas exchange (carbon dioxide in, oxygen out) needed for photosynthesis (Shimazaki et al., 2007). Stomata are present in several plant organs (e.g., leaves, stems, reproductive structures) and are composed of two guard cells, whose changes in shape determine the size of the pore and consequently the rate of gas exchange.

Light and circadian clock control stomata movements. On the one hand, stomata open in the morning in response to blue light and close at the end of the day (Kinoshita, 1999; Kinoshita et al., 2001). On the other hand, stomata functioning is under the influence of outputs of the circadian clock, which regulate the activity of a proton pump (H^+^-ATPase) that generates osmotic pressure in the guard cells: stomata open when guard cells swell due to water intake and close when guard cells shrink due to water loss. Mutations in genes associated with the circadian clock (such as *CCA1*) affect stomata opening/closing cycle, making loss of function mutants unable to anticipate the day/night changes (Hassidim et al., 2017).

Plants grown under LDs present a higher stomatal conductance than those grown under SDs, and this difference remains for a week after changing the conditions from LD to SD (Aoki et al., 2019). Recently, some factors of the photoperiod pathway involved in the control of the floral transition were also shown to regulate stomata functioning, including the florigen. For example, FT has a non-cell autonomous function in flowering time when expressed in leaf vasculature (see previous section on the mobile florigen) but also a novel cell-autonomous role in stomatal opening when expressed in leaf guard cells, where it promotes H^+^-ATPase activity (Kinoshita et al., 2011). Moreover, there is a direct correlation between *FT* levels and the light-induced stomatal opening, being wider when plants grow under LDs than under SDs (Kinoshita et al., 2011). *TWIN SISTER OF FT* (*TSF*), the close homolog of *FT*, not only promotes flowering redundantly with FT, but also stomata opening, as mutations in *TSF* impair stomatal responses (Ando et al., 2013).

Together with the florigens, the floral activators GI and CO are also involved in stomata functioning: while mutations in *GI-CO-FT*/*TSF* suppress stomatal opening induced by light, their overexpression rescues the closed stomata phenotype of mutants defective in the blue light receptors phototropins (Kinoshita et al., 2011; Ando et al., 2013). Likewise, photoperiod is able to change the epigenetic regulation of *SOC1* (Aoki et al., 2019) and the overexpression of the floral integrator *SOC1* in guard cells promote stomata opening (Kimura et al., 2015).

Conversely, ELF3 – a circadian clock associated protein that represses floral activators – negatively regulate stomata opening: the *elf3* mutants exhibit a permanently open stomata phenotype under continuous light. Thus, the circadian rhythm on the stomatal opening regulation may depend at least partially on ELF3 regulation of *FT* (Kinoshita et al., 2011).

From a physiological point of view, the control of stomatal movements mediated by key regulators of the photoperiodic pathway may be beneficial for plants undergoing the floral transition in suboptimal conditions. When grown under water limitation, adult plants might activate the so-called drought escape, which consists of early flowering and accelerated metabolism: open stomata result in increased gas exchange and photosynthetic activity (Shavrukov et al., 2017) to provide more nutrients and energy for the anticipated reproductive development.

## Conservation and Diversification of Flowering Time Genes in Cereal Crops

In the last decades, independent groups demonstrated that members of the PEBP family showing high similarities with the *Ar*abidopsis FT trigger flowering in different plant species (some reviews: Andrés and Coupland, 2012; Wickland and Hanzawa, 2015; Khosa et al., 2021), reinforcing the idea of the existence of a **universal florigen**. For instance, the *FT* orthologs in the most cultivated cereal crops studied so far have been mapped to the *Heading date 3a* (*Hd3a*) locus in rice (Tamaki et al., 2007), *DAYS* TO ANTHESIS 8 (DTA8) locus corresponding to ZEA CENTRORADIALIS 8 (ZCN8) in maize (Danilevskaya et al., 2011; Lazakis et al., 2011; Meng et al., 2011; Guo et al., 2018), *VER*NALIZATION 3 (VRN3) loci corresponding to HvFT1 and TaFT in barley and wheat (Yan et al., 2006; Faure et al., 2007; Kikuchi et al., 2009).

Despite the conserved function of FT-like proteins, cereals have evolved species-specific molecular mechanisms that regulate plant response to photoperiod and modulate time to flowering (also termed **heading date** in rice, barley and wheat). To date, major factors found to control circadian rhythms and photoperiodic flowering in cereals contain the CCT domain – a conserved sequence of 41–43 amino acids named after CONSTANS, CO-like, and TOC1 proteins previously characterized in *Arabidopsis*. These regulatory proteins can be divided into three main families: COL (CO-like), also having one or two Zinc Finger BBX at the N-terminus; PRR, also having a pseudo receiver domain at the N-terminus; CMF (CCT Motif Family), only having a CCT domain at the C-terminus (reviewed in Li and Xu, 2017). While members of the COL and PRR families have been identified in both monocot and dicot species, comparative and phylogenetic analyses of CCT proteins in grasses suggested that the monocot-specific CMF group likely derived from common ancestors of the COL group after the monocot-dicot divergence (Cockram et al., 2012).

Here, we review strategies underlying flowering time in important cereal crops, with a particular focus on the regulatory role of species-specific CCT-domain containing proteins acting upstream of the conserved florigen.

### Photoperiodic Flowering in Cereals of Tropical Origin

The two most cultivated cereal crops worldwide, maize (*Zea mays*) and Asian rice (*Oryza sativa*), derive from wild species grown more than 9000 years ago in tropical and subtropical regions. Precisely, maize was domesticated from *Zea mays* ssp. *parviglumis* (also known as Teosinte) in central Mexico (Matsuoka et al., 2002; Yang et al., 2019) whereas Asian rice was domesticated from Oryza rufipogon in the Yangtze River basin in China (Purugganan, 2014).

In both regions, wild ancestors behaved as SD plants, meaning that flowering is promoted in response to a photoperiod of 12 h light/12 h dark or below this critical daylength. Through centuries of cultivation, maize and rice spread to higher latitudes thanks to the artificial selection of mutant plants that acquired the ability to flower when daytime is above 12 h of light. It follows that natural variation in photoperiodic response has allowed the expansion of cereals of tropical origin to temperate regions characterized by a single LD growing season. Which is the molecular basis of this adaptation?

Unlike the Arabidopsis CO, the rice homolog of CO – called Heading date 1 (Hd1) – promotes flowering under inductive SDs but delays it under LDs (Yano et al., 2000; Izawa et al., 2002; Kojima et al., 2002). In non-inductive conditions, Hd1 protein interacts with Grain number plant height and heading date 7 (Ghd7), a floral repressor belonging to the CMF family that is active when the daylength exceeds 13.5 h light (Xue et al., 2008; Itoh et al., 2010; Nemoto et al., 2016). Ghd7 delays the reproductive phase under LDs by repressing *Early heading date 1* (*Ehd1*), which encodes a B-type response regulator that activates the florigen in the leaf (Doi et al., 2004; Itoh et al., 2010). Ghd7 also regulates overall plant growth and grain yield: a prolonged vegetative phase under LD correlates with increased plant biomass and seed production (Xue et al., 2008). Likewise, the PRR-like protein Days to heading 7 (DTH7/OsPRR37) also delays the floral transition under LD by repressing the floral activator *Ehd1*, leading to increased plant height and grain production (Gao et al., 2014).

Like the Arabidopsis CO, Hd1 physically associates with rice NF-Y proteins: the formation of heterotrimeric complexes containing NF-YB and NF-YC subunits is instrumental for binding to specific regulatory sequences of the downstream target gene *Hd3a* (Goretti et al., 2017; Shen et al., 2020). Intriguingly, OsPRR37 also seems to interact with heterodimers formed by NF-YB and NF-YC (Goretti et al., 2017). Thus, NF-Y proteins play key roles for the correct functioning of the rice CCT-type regulators, which control important traits related to agronomic performance by negatively regulating heading date under LD.

It’s important to highlight that genetic variation at loci encoding major floral repressors underlies phenotypic variation in photoperiod responsiveness of varieties adapted to temperate regions. In fact, rice accessions cultivated in Europe harbor loss of function mutations in Hd1/Gh7/OsPRR37 that fail to repress the floral transition under LDs (Gómez-Ariza et al., 2015; Goretti et al., 2017).

Recent findings further support the conserved function of CCT-type floral repressors in maize. Indeed, *ZmCTT9* and *ZmCCT10* – the maize orthologs of *Ghd7* – map to *DAYS TO ANTHESIS 9* (*DTA9*) and *DTA10* loci, two of the most important QTLs controlling flowering time in this cereal crop (Guo et al., 2018). Over time, the accumulation of polymorphisms in key *DTA* loci, caused by intense transposon activity, has determined alterations of photoperiod sensitivity in selected maize cultivars. Specifically, maize accessions cultivated in Northern and Southern America carry defective alleles of *ZmCTT9* and *ZmCCT10*, which correlate with activation of the florigen ZCN8 and consequently accelerated flowering under LD (Yang et al., 2013; Huang et al., 2017; Guo et al., 2018).

**FIGURE 1:**
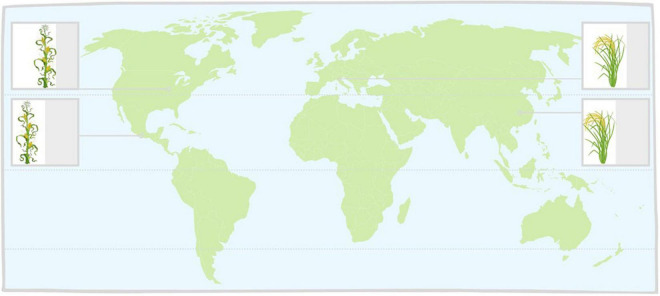
Adaptation of cereal growth to different latitudes. Maps showing the expansion of maize **(left)** and rice **(right)** cultivation outside their region of origin. The selection of mutation in genes encoding CCT-type floral repressors that alter photoperiodic response allowed activation of the florigen under non inductive LDs.

To sum up, the selection of genetic variants with reduced activity of LD floral repressors has allowed the expansion of rice and maize cultivation outside tropical and subtropical regions, to a wider range of growing areas at higher latitudes and daylength above 12 h of light (Figure 1).

### Photoperiodic Flowering in Temperate Cereals

Barley (*Hordeum vulgare*) and wheat (*Triticum aestivum*), the founders of agriculture in the old world, derive from wild ancestors grown 10,000 years ago in the middle east (discussed by Haas et al., 2019). Over the centuries, the cultivation of these grain crops had expanded from the Fertile Crescent (Latitude N 38) to temperate regions in Europe and Asia with similar latitude, mainly across the East-West axis, in which cold winter is followed by warm LDs and hot summer is followed by cool SDs.

Temperate cereals behave as quantitative LD plants, meaning that flowering is delayed when days are short but promoted when days become longer. Specifically, floral induction occurs in spring when plants reach a threshold determined by a certain number of LDs. However, seasonal changes in the photoperiod do not directly affect the timing of the floral transition, which is under the influence of vernalization, but rather the initiation of reproductive structures at the shoot apex in barley and wheat (reviewed by Hyles et al., 2020).

Modern winter varieties of barley are sown in autumn and usually accelerate flowering in spring (Turner et al., 2005). This response is mediated by Photoperiod-H1 (Ppd-H1), a barley circadian clock associated protein similar to Arabidopsis PRR7 that positively regulates the florigen *HvFT1* under LDs (Turner et al., 2005). Upon floral induction, Ppd-H1 also accelerates early and late phases of reproductive growth by activating floral homeotic genes (Digel et al., 2015).

Genetic variation at *Ppd-H1* associate with phenotypic variation in the timing of reproductive development. Precisely, many recessive alleles contain Single Nucleotide Polymorphisms (SNPs) responsible for amino acid changes in conserved domains that impair Ppd-H1 protein function, including the SNP79 (G- > T, described by Turner et al., 2005). Varieties carrying functional *Ppd-H1* alleles (G at position 79) differentially express *HvFT1* in response to daylength, leading to accelerated flowering under LD but delayed flowering under SD (Turner et al., 2005). Conversely, varieties carrying defective *ppd-H1* alleles (G- > T SNP79) flower late even when grown under LD, because of reduced expression of *CO-like* genes and *HvFT1* (Turner et al., 2005).

In European barley accessions, the frequency of two alleles at SNP48 (C- > T, described by Jones et al., 2008) correlates with clinal variation in flowering phenotype. Specifically, the functional *Ppd-H1* allele (C at position 48) predominates in Southern Europe, characterized by a short growing season; in these regions, cultivars accelerate flower development in spring and seeds reach maturity before terminal drought in summer. By contrast, the non-functional *ppd-H1* allele (C- > T SNP48) predominates at Northern latitudes, characterized by a long growing season; in these regions, accessions with prolonged vegetative phase also show increased biomass accumulation and higher grain yield at harvesting. Thus, polymorphisms at *Ppd-H1* underly adaptation to different environmental conditions and largely explain latitude-dependent geographical distribution of barley varieties, at least in Europe.

In the hexaploidy genome of wheat, three orthologs of the barley Ppd-H1 (Ppd-A1, Ppd-B1, and Ppd-D1) were identified and Ppd-D1 shown to have the greatest contribution to the regulation of flowering (Guo et al., 2010; Würschum et al., 2018).

A semi-dominant mutation caused by a 2 kb deletion in the promoter region of *Ppd-D1* converts a LD plant into a day neutral plant by altering the diurnal expression pattern of *Ppd-D1.* Under SD, the peak of *Ppd-D1* transcription shifts from the light to dark phase, causing induction of the florigen *TaFT1* and promotion of flowering regardless of daylength (Beales et al., 2007). Additional polymorphisms in the regulatory sequences of *Ppd-D1* were identified in a panel of 500 common wheat varieties cultivated worldwide (Guo et al., 2010). To a large extent, accessions with higher *Ppd-D1* expression (under LD) flower earlier than those with lower *Ppd-D1* expression. Similar to barley, photoperiod sensitive accessions delay flowering under SD while photoperiod insensitive varieties initiate the reproductive phase regardless of daylength (Guo et al., 2010).

To recap, PRR proteins promote flowering under LD in temperate cereals. Mutations in the coding sequence of *Ppd-H1* largely explain alterations in the timing of flower development in barley, whereas mutations at regulatory regions of *Ppd-D1* gene contribute to variation in the photoperiodic responsiveness in wheat. Together with vernalization requirements, changes in the activity of Ppd proteins have determined the adaptation of temperate cereals to different geographic regions, thus allowing their cultivation in a wide range of agroecosystems.

## Photoperiodic Control of Seasonal Growth in Perennial Plants

In contrast to annual herbaceous plants that complete their life cycle and die within a single year, perennial plants can live for several growing seasons - continuously in warm climates and discontinuously in temperate climates.

Photoperiodic cues and changes in ambient temperature greatly influence the seasonal behavior of tree populations growing in temperate regions (Wareing, 1956). Indeed, woody perennials display active growth in response to lengthening days in spring but growth cessation in response to shortening days at the end of the summer. Below a critical photoperiod in autumn, the apical buds formed enter a dormant state (completed in 2–3 months) to withstand very low temperatures and reduced light in winter (Garner and Allard, 1923). Generally, trees pause their vegetative growth in the cold season but resume it the next spring (Garner and Allard, 1923).

In woody perennials, the production of flower buds can take several years as plants have to undergo the transition from the juvenile to the adult phase to acquire the competence to form reproductive structures. Following first-time flowering, trees flower every year throughout their life span. Thus, the perennial growth habit consists of annual cycles of growth and dormancy as well as annual cycles of vegetative and reproductive development, depending on the season.

Surprisingly, annuals and perennials share similar molecular mechanisms underlying photoperiod response, despite the substantial differences in their life history traits. So far, most of the studies aimed at unraveling the photoperiodic control of seasonal growth in trees have been conducted in species belonging to the *Populus* genus, which have also served as models to investigate the function of putative orthologs of Arabidopsis flowering time regulators.

In *Populus trichocarpa* (California poplar), two FT-like genes have been identified: PtFT1 and PtFT2 (Böhlenius et al., 2006*;* Hsu et al., 2006). Their transcripts accumulate in different domains and seasons: the former in stem and apical buds in late winter, the latter in leaves in late spring-early summer (Böhlenius et al., 2006; Hsu et al., 2006, 2011).

Although constitutive expression of each of the two *FT* paralogs caused early flowering in transgenic poplars (Böhlenius et al., 2006; Hsu et al., 2006), inducible expression of *FT1* and *FT2* (driven by Heat shock promoter) resulted in different phenotypes, pointing to a possible sub-functionalization of *FT-like* genes (Hsu et al., 2011). Indeed, transient induction of *FT1* (but not *FT2*) under SD at low temperature promotes the formation of reproductive structures within 1 month from the heat treatment (Hsu et al., 2011). By contrast, transient induction of *FT2* (but not *FT1*) promotes vegetative growth under LD and inhibits growth cessation under SD. These findings indicate that *FT1* activation in winter is required for the onset of reproductive structures whereas *FT2* activation in spring promotes vegetative growth (Hsu et al., 2011).

Moreover, *FT2* suppression in autumn mediates growth cessation in response to environmental limitations (Hsu et al., 2011). Actually, the critical daylength required to induce growth cessation is longer in trees growing at northern latitudes compared to those at southern latitudes, implying that alterations in photoperiodic response might have determined adaptation of tree populations to different environments (Frewen et al., 2000; Böhlenius et al., 2006).

Supporting the sub-functionalization of the two FT paralogs in poplar, large scale expression analysis of transgenic plants mis expressing *FT-like* genes revealed differences in the molecular networks controlled by FT1 and FT2: while genes involved in reproductive development act downstream of FT1 at winter onset, genes involved in vegetative growth and stress response act downstream of FT2 (Hsu et al., 2011).

To summarize, the differential expression of *FT* paralogs in contrasting seasons (*FT1* in late winter, *FT2* in mid spring) reinforces a diverged regulatory function in annual cycles of vegetative and reproductive growth (Hsu et al., 2011). In woody perennials, FT-like proteins appear to integrate key environmental signals (photoperiod and ambient temperature) to promote seasonal growth under warm LD in spring but increase survival (via dormancy) under cold SD in winter.

Factors showing similarity to components of the Arabidopsis photoperiodic pathway seem to have a conserved function in trees.

Downstream of FT-like proteins, poplar *FD Like 1* (*FDL1*) and *Like AP1* (*LAP1*) genes also show continuous upregulation during bud development (Ruttink et al., 2007). Under LD, FT2, and FDL1 promote vegetative growth in spring by activating *LAP1*. As days become shorter, the downregulation of these genes allows growth cessation (Böhlenius et al., 2006; Hsu et al., 2006; Ruttink et al., 2007).

Upstream of FT-like proteins, two paralogs of GI have been identified in the hybrid *P. tremula* × *P. tremuloides* and shown to play a critical role in the regulation of seasonal growth (Ding et al., 2018). Silencing lines downregulating both *PttGI* and *PttGIL* initiate growth cessation and formation of apical buds even under LDs, and these processes occurred within 1 week in plants shifted from LD to SD, indicating a hypersensitive response to photoperiod (Ding et al., 2018). Conversely, plants overexpressing *PttGI* and *PttGIL* initiate growth cessation at least 1 month after the shortening of daylength, indicating a decreased sensitivity to photoperiod (Ding et al., 2018). In these transgenic plants, alterations of the critical daylength that induces growth cessation and bud set correlate with mis regulation of *PttFT2*, which is downregulated in *GI* silencing lines and upregulated in *GI* overexpression lines (Ding et al., 2018).

Additional molecular and biochemical analyses revealed that PttGIs positively regulate *PttFT2* by directly binding its promoter, likely by forming complexes with FKF1-like and CDF-like proteins (Ding et al., 2018). Thus, GI-like proteins in *Populus* trees act as strong transcriptional activators of *FT*, largely independently of CO-like proteins.

The effect of photoperiod on seasonal growth has also been studied in other perennials. In the non-woody perennial leafy spurge (*Euphorbia esula* L.), crown buds (i.e., adventitious buds located on the underground stem) show downregulation of a *FT-like* gene and induction of *DORMANCY ASSOCIATED MADS-BOX* (*DAM*) genes (Horvath et al., 2008), which share similarities with the Arabidopsis *SVP*. DAMs play key regulatory roles in the maintenance of dormancy in *Euphorbia* and might be acting as repressor of the *FT-like* gene (Horvath et al., 2008).

Photoperiod controls seasonal growth in angiosperms as well as in gymnosperms (Heide, 1974). Several studies done in the conifer *Picea abies* (Norway spruce) uncovered the presence of two genes encoding PEBP proteins that share similarities with the Arabidopsis florigen FT and the anti-florigen TERMINAL FLOWER 1 (TFL1, Shannon and Meeks-Wagner, 1991), and so renamed *PaFTLs* (Karlgren et al., 2011).

Gene expression analyses in wild-type spruce showed that *PaFTL1* mRNA accumulates after the winter in male reproductive structures whereas *PaFTL2* mRNA accumulates in shoots in response to decreasing daylength, coinciding with growth cessation and bud set (Gyllenstrand et al., 2007; Karlgren et al., 2011; Klintenäs et al., 2012). Interestingly, overexpression of *PaFTL1* do not cause morphogenetic effects in transgenic spruce, whereas constitutive expression of *PaFTL2* in tissue cultures caused growth arrest and death within 6 months (Klintenäs et al., 2012).

Additional genetic and physiologic studies revealed that (latitudinal) clinal variation in the photoperiodic control of growth cessation associates with genetic variation in *PaFTL2* promoter and one variant of the PaGI protein in Norway spruce as well as Siberian spruce (Chen et al., 2012, 2014).

To conclude, FT/TFL1-like proteins might have retained a general function as growth regulators in gymnosperm and acquired a specific function as flowering time regulators in angiosperms. The knowledge about these mechanisms will be very useful in the years to come. With shifting seasons and climate conditions, new breeding programs need to be launched to develop species adapted to changing conditions and to keep up with the increasing demand on forest resources.

## Photoperiodic Control of Growth Above and Below Ground

In addition to flowering in annual plants and growth habit in perennial plants, photoperiod also controls vegetative propagation in relevant food plants such as potato (*Solanum tuberosum*), the most important non-cereal crop for direct human consumption, and strawberry (*Fragaria x ananassa*), one of the most widely consumed berry crops in the world. In commercial plant varieties, asexual reproduction through vegetative structures represents an essential propagation strategy that allows producing identical daughter plants that keep the desirable characteristics of the mother plant.

### Flowering Versus Tuberization in Potato

Potato (*Solanum tuberosum*) is the fourth most cultivated food plant globally after maize, rice and wheat (FAO 2019^1^). Its domestication from wild *Solanum* species started 8,000–10,000 years ago in the Andean highlands, an arid region 3,000–4,500 m above sea level characterized by cold temperatures, saline soils, and high solar radiation (Zimmerer, 1998; Spooner et al., 2005; Sukhotu and Hosaka, 2006).

Diploid landraces (2n = 24) underwent autopolyploidization and gave origin to the cultivated tetraploids (2n = 48) belonging to the *Solanum tuberosum* group Andigena (Hardigan et al., 2017; Gutaker et al., 2019). Later, the cultivation of potato expanded to highland equatorial regions and to southern latitudes. In Argentina and Chile, *Solanum tuberosum* group Chilotanum diversified from its upland progenitors to adapt to LD conditions (Ghislain et al., 2009). Together with wild species, these landraces have substantially contributed to the development of modern varieties (known as Neo-tuberosum, 2n = 48) that are grown globally in a wide environmental range (Hardigan et al., 2017).

The high heterozygosity of the potato genome, which resulted from wild species introgression and polyploidy, has hampered classical genetic studies, and favored vegetative propagation through **tubers**, storage organs growing in the soil that also bear vegetative buds for the following season. Potato plants can use asexual reproduction via tuberization as well as sexual reproduction through flowering. Nevertheless, tuber initiation and flower development seem to be antagonistic processes (Plantenga et al., 2019).

In potato, photoperiod controls the formation of vegetative structures that differentiate from underground stems called stolons (Batutis and Ewing, 1982), which form new shoots above ground under LDs but form tubers below ground under SDs. Once formed, dormancy of the apical meristem is induced before winter.

The considerable genetic variation in potato accessions, a result of centuries of domestication and diversification (Hardigan et al., 2017; Li Y. et al., 2018), has led to a large phenotypic variation in responsiveness to photoperiod. Indeed, tuberization is promoted by SDs in all species but this process can also occur under LDs in modern varieties (Rodríguez-Falcón et al., 2006). For example, in the obligate SD varieties of the *Andigena* group, tuberization is induced under 12 h light/12 h dark but is completely abolished above this critical daylength (reviewed by Jackson, 1999). By contrast, selected varieties growing in temperate regions trigger the transition from stolon to tuber under LDs. Thus, alteration of photoperiodic response represents a key adaptive trait also in potato (Morris et al., 2014).

The link between potato tuberization and daylength was described almost a century ago (Garner and Allard, 1923) but the main tuber-inducing molecule was characterized only 10 years ago (Navarro et al., 2011). The so-called **tuberigen** is encoded by *SELF PRUNING 6A* (*StSP6A*), named after the tomato florigen SELF-PRUNING (homolog of FT).

Although three additional *FT-like* genes (*StSP5G*, *StSP5G-like*, and *StSP3A*) were identified in the potato genome (Xu et al., 2011), only *StSP6A* showed expression in leaves that correlated with tuber formation at the stolon tip in SDs (Navarro et al., 2011). Since *StSP6A* is also transcribed in stolons, a relay mechanism could regulate *StSP6A* expression and sustain the production of the tuberigen in stolons (Navarro et al., 2011).

Several lines of evidence suggest that StSP6A functions as the main tuberization signal that travels from the leaves to the target meristem (i.e., the tip of the stolon below ground) whereas the related StSP3D as regulator of flowering (Navarro et al., 2011). Supporting their distinct roles, transgenic potato plants overexpressing *StSP6A* form tubers even under non-inductive LDs while *StSP3D* silencing lines flower late but do not display alterations in tuber formation (Navarro et al., 2011).

It was originally hypothesized that tuberization could impair flowering by serving as sink for photosynthates. However, a recent study reported that it is the activity of StSP6A that promotes tuber formation below ground but inhibits flower development above ground (Plantenga et al., 2019). Indeed, *StSP6A* silencing lines display decreased tuberization but increased flower bud development when grown under SDs (Plantenga et al., 2019).

As photoperiod controls tuber initiation, several groups have explored the possible role of homologs of Arabidopsis flowering time regulators in this process.

Among the three *CO-like* genes (*StCOL1*/*StCOL2*/*St*COL3, located in tandem array on chromosome 2) identified in the potato genome (Abelenda et al., 2016; Ramírez Gonzales et al., 2021), *StCOL1* displays the highest transcription in leaves and preferential accumulation under LDs when the peak of expression coincides with light (Navarro et al., 2011; Abelenda et al., 2016; Ramírez Gonzales et al., 2021). *StCOL1* downregulation in transgenic *RNAi* lines accelerated tuberization (González-Schain et al., 2012) and the constitutive expression of Arabidopsis *CO* in Andigena potato resulted in delayed tuberization under SD (Martínez-García et al., 2002). Taken together, these findings indicate that CO functions as suppressor of stolon-to-tuber transition in potato, likely by repressing *the tuberigen* under non-inductive daylengths.

Detailed expression analysis of *FT-like* genes (Abelenda et al., 2014) in *StCOL1* silencing lines revealed upregulation of the tuber-inducing *StSP6A* but down-regulation of *StSP5G*, which is normally highly expressed in leaves under LDs but decays under SDs (Navarro et al., 2011). Additional molecular studies demonstrated that StCOL1 directly activates *StSP5G* under LDs by binding to a conserved TGTGGT DNA motif (similar to the CORE bound by AtCO) in its regulatory regions. Upon activation, StSP5G represses tuberization under non-inductive conditions by negatively regulating *StSP6A* transcription. Thus, the two FT paralogues StSP6A and StSP5G act antagonistically during tuber formation in potato. As supporting evidence, RNAi lines downregulating *StSP5G* showed *StSP6A* upregulation in leaves and accelerated tuberization under LD (Abelenda et al., 2016).

Another crucial factor involved in the regulation of tuberization is StCDF1 (Kloosterman et al., 2013), the homolog of the *CO* repressors AtCDFs (Fornara et al., 2009). Allelic variation at *StCDF1* underlies a major QTL controlling yield and other traits related to maturity – such as the duration of the plant life cycle, the onset of senescence, and timing of tuber induction under LDs (Kloosterman et al., 2013). Very late maturing genotypes harbor functional *StCDF1* alleles encoding full length proteins, whereas very early maturing genotypes harbor defective *StCDF1* alleles (caused by the insertion of transposable elements in the 3′end of the gene) that encode deleted versions lacking part of the C-terminus (Kloosterman et al., 2013). In Arabidopsis, the C-term domain is essential for interaction with the GI-FKF1 complex that mediates CDFs degradation (Imaizumi et al., 2005; Sawa et al., 2007). Protein-protein interaction studies confirmed that the full length StCDF1 (encoded by late alleles) physically associates with StGI1 and StFKF1, while truncated StCDF1 (encoded by early alleles) fails to associate with its regulators and evades StFKF1-mediated ubiquitination. Thus, the circadian clock proteins StGI and StFKF1 control the accumulation of StCDF1 by binding its C-terminus: while full length StCDF1 displays a peak of protein abundance at midday, truncated proteins accumulate constantly during the day (Kloosterman et al., 2013).

**FIGURE 2:**
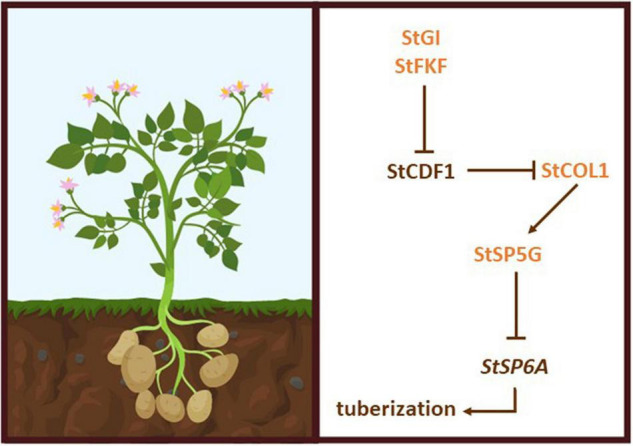
Regulatory mechanism underlying tuber formation in potato. Right, components of the genetic pathway controlling tuberization. The tuberigen StSP6 is indirectly activated by StCDF1 and repressed by StCOL1 through StSP5G.

Recent studies demonstrated that StCDF1 represses *CO-like* genes by directly binding DOF consensus sequence in their promoter regions (Ramírez Gonzales et al., 2021). Therefore, StCDF1 promotes tuber formation by indirectly suppressing the tuber repressor *StSP5G* (via *StCOL1/2*) and activating the tuber inducer *StSP6A* (Figure 2). Indeed, transgenic plants overexpressing *StCDF1* show strong upregulation of *SP6A* and downregulation of *StSP5G* and *StCOL1* (Kloosterman et al., 2013). Interestingly, overexpression of shorter *StCDF1* variants in *Andigena* potato does not affect flowering but accelerates tuberization under LD and senescence, leading to a shorter life cycle (Kloosterman et al., 2013).

A Genome-Wide Association Study (GWAS) carried out on a pool of 83 potato cultivars reinforced the correlation between polymorphisms at *StCDF1* locus and maturity phenotype (Kloosterman et al., 2013). Likewise, the analysis of several *StCDF1* haplotypes confirmed that most modern LD-adapted varieties carry defective *StCDF1* alleles encoding shortened forms that escape degradation by the proteasome mediated by circadian clock proteins (Hardigan et al., 2017). This diurnal deregulation results in increased stability of StCDF1 protein, which leads to constitutive repression of *StSP5A* and accumulation of *StSP6A* under LDs. Thus, naturally occurring structural variants of StCDF1 account for the adaptation of potato varieties to higher latitudes characterized by LD summer and SD winter.

Besides components of the photoperiodic pathway, gibberellins also have a role in the formation of tubers. Treatments with exogenous GA promote stolon elongation but inhibit tuberization (Kumar and Wareing, 1974; Xu et al., 1998). Conversely, application of a GA biosynthesis inhibitor allows tuberization in non-inducing LDs (Jackson and Prat, 1996). More recently, several GA metabolism genes have been shown to be involved in different stages of the tuberization process (Xu et al., 1998; Jackson et al., 2000; Kloosterman et al., 2007; Bou-Torrent et al., 2011).

Phytochromes are also involved in the photoperiodic control of tuberization. Among the five phytochrome genes identified in the potato genome, StPHYB and StPHYF play the most prominent role in the inhibition of tuberization in response to LDs (Jackson and Prat, 1996; Jackson et al., 1998; Zhou et al., 2019). In fact, silencing of *StPHYB* or *StPHYF* caused tuberization in LD in a graft-transmissible manner (Jackson and Prat, 1996; Jackson et al., 1998; Zhou et al., 2019). StPHYB and StPHYF might interact to form heterodimer and stabilize the StCOL1 protein (Abelenda et al., 2016; Zhou et al., 2019).

The homeodomain protein StBEL5 is another crucial transcription factor involved in the regulation of tuber formation: *StBEL5* overexpression promotes tuberization under LDs (Banerjee et al., 2007), whereas *StBEL5* silencing reduces tuberization (Sharma et al., 2016). Although *StBEL5* expression itself does not seem to be affected by photoperiod (Chatterjee et al., 2007), SDs result in increased *StBEL5* transcript levels and facilitated movement of its mRNA from leaves to stolons (Lin et al., 2013), where it is translated and functional (Banerjee et al., 2007, 2009).

Last, microRNAs *miR156* and *miR172* are also involved in tuberization. Overexpression of *miR156* reduces normal below-ground tuberization but leads to aerial tuber formation in potato (Bhogale et al., 2014) and even in non-tuberizing tomato (Eviatar-Ribak et al., 2013).

Overexpression of *miR172* in Andigena potato promotes tuberization in LDs, likely through downregulation of an *AP2-like* gene and upregulation of *StBEL5* (Martín et al., 2009).

Both these tuberization phenotypes caused by overexpression of microRNAs are graft-transmissible (Martín et al., 2009; Bhogale et al., 2014), showing that they could also be part of mobile tuberization signal together with StSP6A.

### Flowering Versus Runner Formation in Strawberry

In strawberry, axillary meristems have three possible destinies: they can remain dormant or develop into either a crown branch (a new leaf rosette which may eventually produce an inflorescence) or a runner, horizontal elongated stem that grows above the ground (Darrow, 1966). This means that for a particular axillary meristem, flowering and runnering are mutually exclusive (Hytönen et al., 2009; Mouhu et al., 2013).

In general, conditions that promote flowering decrease the number of runners, and many strawberry cultivars develop runners under LDs when flowering is not induced (Hartmann, 1947; Konsin et al., 2001; Hytönen et al., 2004).

Strawberry varieties can be classified in two groups depending on their photoperiodic behavior: most are seasonal SD-flowering plants that readily produce runners in LDs, while others are ever-flowering plants that develop reproductive structures preferentially under LDs but produce very few runners or are completely runnerless (Darrow and Waldo, 1934; Nishiyama and Kanahama, 2002; Mouhu et al., 2009).

Given the agronomic relevance of runner formation for asexual reproduction of commercial strawberries (Hytönen et al., 2009), molecular studies have been employed to understand the regulatory mechanisms controlling this process. New knowledge could be used in breeding programs for crop improvements.

Strawberry species belong to the *Fragaria* genus and vary in their ploidy, ranging from diploid to decaploid (Hummer and Hancock, 2009; Edger et al., 2019). Since the octoploid genome of the widely cultivated garden strawberry (*Fragaria x ananassa)* makes genetic analysis impractical, research has been performed mostly in diploid woodland strawberry (*Fragaria vesca)*.

Gibberellins are essential for the differentiation of vegetative structures from axillary meristems: runner formation is promoted by treatments with exogenous GA even in runnerless varieties (Thompson and Guttridge, 1959; Tenreira et al., 2017), but prevented by chemical inhibition of GA biosynthesis (Guttridge and Thompson, 1964; Ramina et al., 1985; Hytönen et al., 2009). Interestingly, runnerless accessions harbor a deletion in the GA biosynthetic gene *FvGA20ox4*, which is mostly expressed in axillary meristems and developing runners (Tenreira et al., 2017). Furthermore, a genetic screen conducted with a runnerless woodland strawberry accession led to the identification of a mutation in a gene called *Suppressor of Runnerless* (*SRL*) (Caruana et al., 2018), which was renamed *FvRGA1* because of its high similarity with *DELLA* gene, that caused constitutive runner formation (Kang et al., 2013). A later study showed that *FvRGA1* silencing induces the formation of runners in non-runnering varieties (Li W. et al., 2018).

Runner formation in strawberry also shares crucial components with the photoperiodic pathway that control flowering in Arabidopsis.

Under LDs, FvCO is required for the expression of *FvFT1* (Rantanen et al., 2014; Kurokura et al., 2017), although the interplay between FvCO and FvFT1 is not exactly the same as in Arabidopsis, since *FvCO* mRNA is expressed at different times during the day. Upon activation, FvFT1 induces *FvSOC1*, which promotes the expression of GA biosynthetic genes including *FvGA20ox4* (Andrés et al., 2021). Consequently, accumulation of GA causes the degradation of the FvRGA1/SRL, leading to runner formation. Interestingly, treatments with GA biosynthesis inhibitors block runner formation even in plants overexpressing *FvSOC1* (Mouhu et al., 2013).

Under SDs, *FvFT1* is repressed, FvSOC1 is not active and GAs do not accumulate; this means that FvRGA1/SRL is not degraded and able to repress runnering.

Flowering is also inhibited by *FvSOC1* through *FvTFL1*, which displays similarities with the Arabidopsis antiflorigen. As supporting evidence, perpetual flowering accessions carry mutations in the *FvTFL1* gene (Koskela et al., 2012; Mouhu et al., 2013) that impair its function as floral repressor.

## Concluding Remarks

**FIGURE 3:**
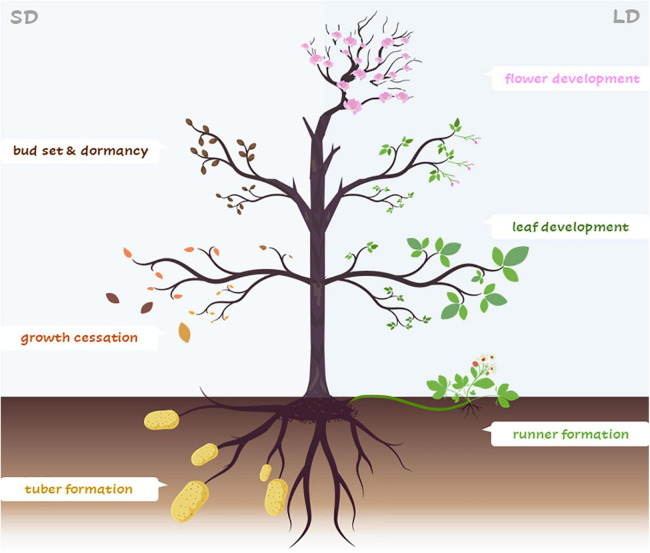
Frankenstein plant. Representation of an imaginary plant showing specific developmental processes controlled by similar components of the photoperiodic pathway in different plant species. Under LDs **(right)** leaf and flower development, runner formation (specific for strawberry). Under SD **(left)** bud set/dormancy and growth cessation (perennial trees), tuber formation (specific for potato).

In this review, we have highlighted the function of those components of the photoperiod pathway that happen to regulate different developmental processes in different plant species (Figure 3 and Supplementary Table 1). The genetic pathways that control plant development in response to photoperiodic cues presented here converge on the regulation of members of the PEBP family highly similar to the florigen FT (Table 1).

**TABLE 1:** Role of FT-like genes in photoperiod-controlled processes discussed in the review.

Organism	Gene name	Role of FT-like genes in photoperiod-controlled processes
*Arabidopsis thaliana*	*FT, TSF*	Positive regulation of flowering under LD and control of stomata opening
*Oryza sativa*	*Hd3a, RFT1*	Positive regulation of flowering under SD Positive regulation of flowering under LD
*Zea mays*	*ZCN8*	Positive regulation of flowering (days to anthesis)
*Hordeum vulgare*	*HvFT1*	Positive regulation of flowering (heading date)
*Triticum aestivum*	*TaFT1*	Positive regulation of flowering (heading date)
*Populus trichocarpa*	*PtFT1 PtFT2*	Promotion of growth cessation and bud formation. Promotion of bud burst
*Picea abies*	*PaFT4*	Promotion of growth cessation and bud set
*Solanum tuberosum*	*StSP6A*	Positive regulation of tuberization
*Fragaria vesca*	*FvFT1*	Positive regulation of runner formation

Decades of research in the model species *Arabidopsis thaliana* led to the identification and functional characterization of hundreds of regulatory genes acting upstream of the florigen FT (schematically reported in FLOR-ID, Bouché et al., 2016). Nevertheless, little is known about the regulation of the antiflorigen TFL1 (Fernández-Nohales et al., 2014; Serrano-Mislata et al., 2017), especially at the transition from the vegetative to the reproductive phase.

The antagonism between FT and TFL1 at the shoot apex might represent a brake to slow down the floral transition and avoid accelerated flower development when conditions are not optimal for reproductive success.

According to a recent review by Périlleux et al. (2019), the influence of TFL1 goes beyond the control of flowering time: TFL1 also regulates multiple developmental processes throughout the plant life cycle such as the juvenile to adult phase change, shoot growth and inflorescence architecture. Likewise, the action of FT-like proteins greatly impacts several life history traits in different plants, from Arabidopsis to annual cereals and perennial trees, suggesting that PEBPs may play a general function as plant growth regulators.

In the plant species examined here, the GRNs converging on FT-like factors regulate specific stages of plant development in response to daylength. Intriguingly, genetic variation in components of the photoperiod pathway underlies the phenotypic variation that has allowed plant adaptation to new environments that differ from those of the site of origin.

Many polymorphisms described thus far impair the function of regulators acting upstream of *FT-like* genes, such as loss of function mutations in floral repressors. This likely represents a “survival strategy” to preserve the functionality of PEBPs as fundamental growth regulators and only fine-tune their activity in response to changes in external conditions.

In cereals, the domestication syndrome encompasses a set of changes in natural populations affecting the architecture of vegetative and reproductive organs (e.g., prostrate to erect growth, seed shattering). Instead, the diversification phase relied on the selection of novel varieties that better adapted to new agroecosystems. Noteworthy, genetic diversity in loci encoding circadian clock proteins largely accounts for phenotypic diversity in plant response to photoperiod, which has contributed to adjust the reproductive phase to different environmental conditions and ultimately has favored the cultivation of cereals outside their area of domestication. In fact, the huge genetic variability underlying photoperiod sensitivity has facilitated the reproductive success of cultivated cereals in tropical, subtropical and temperate regions. As the regulation of the floral transition in cereals has influenced both grain yield and adaptive growth, it can be considered one of the most important agronomic traits to obtain improved varieties adapted to stressful conditions arising from changing climates.

In the same manner, selection of allelic variants in the genes responsible for photoperiodic control of potato tuberization has allowed its widespread cultivation at different latitudes and climates, releasing productivity from the constraints of its genetic adaptation to its natural environment. Strawberry is yet another example of a crop in which classical breeding has been based on the alteration of similar GRNs controlling photoperiodic responses, prior to any knowledge of the molecular interactions between its components.

In perennials, changes in photoperiod regulate the critical growth status for tree survival: active growth in warm LDs and growth cessation in cold SDs. The onset of both, to grow and stop growing, is controlled by *FT-like* genes.

In other non-woody perennials, FT orthologs also regulate growth and dormancy; as their role in the control of vegetative development may precede their involvement in floral induction, flowering could also be perceived as a growing period.

## Future Perspectives

In the face of climate change, it’s extremely important to increase our knowledge on the interaction between the photoperiod pathway and other environmental conditions such as ambient temperature or water availability.

It is well established that plants alter their developmental processes to adapt to fluctuating temperatures. For example, hypocotyl elongation and flowering time in Arabidopsis are controlled by the photoperiod but are also greatly influenced by ambient temperatures. Indeed, warmer temperatures promote hypocotyl growth and accelerate flowering. Also, low watering conditions promote precocious flowering, mainly through anticipated activation of the florigen *FT* (Riboni et al., 2013).

Interestingly, overexpression of the main tuberigen *StSP6A* in transgenic potato has been used to increase tuber production under drought and heat stress conditions that are known to negatively impact tuber yield and quality (Lehretz et al., 2021).

Altogether, these studies further highlight the connection between photoperiod responses and other environmental factors as well as the potential of components of the photoperiod pathway as targets for genetic improvement in important plant species.

## Author Contributions

All authors contributed to the article and approved the submitted manuscript. PN and UC prepared the table. MO, IC, and UC ideated figures. MO and SP designed the structure of the review.

## Conflict of Interest

The authors declare that the research was conducted in the absence of any commercial or financial relationships that could be construed as a potential conflict of interest.

## Publisher’s Note

All claims expressed in this article are solely those of the authors and do not necessarily represent those of their affiliated organizations, or those of the publisher, the editors and the reviewers. Any product that may be evaluated in this article, or claim that may be made by its manufacturer, is not guaranteed or endorsed by the publisher.

## Footnotes

1
http://www.fao.org/faostat

## Supplementary Material

Supplementary Table 1 List of genes involved in the regulation of different developmental processes in response to photoperiod.
